# Delayed Diagnosis of Primary Sinonasal Mucosal Melanoma

**DOI:** 10.7759/cureus.113897

**Published:** 2026-08-03

**Authors:** Deysi Olivares-Solis, Rafael A Mejia Jhong, Gustavo G Soto Rojas, Zheleny Claudio Ramos

**Affiliations:** 1 Dermatology, Hospital Augusto Hernandez Mendoza, Ica, PER; 2 Pathology, Hospital Augusto Hernandez Mendoza, Ica, PER; 3 Otoryngology, Hospital Regional de Ica, Ica, PER

**Keywords:** epistaxis, melanoma, mucosal malignant melanoma, nasal mucosa, sinonasal tumor

## Abstract

Primary sinonasal mucosal melanoma (SNMM) is a rare and highly aggressive malignancy that is frequently diagnosed at advanced stages because of its nonspecific presentation. We report the case of a 70-year-old Peruvian woman who presented with a six-month history of recurrent unilateral epistaxis refractory to direct compression, chemical cauterization, and nasal packing, associated with progressive nasal obstruction, facial pain, foul-smelling nasal discharge, and significant weight loss. Nasofibrolaryngoscopy revealed a friable pigmented mass arising from the left nasal cavity. Computed tomography demonstrated a lesion originating from the inferior turbinate without evidence of adjacent bone invasion. Histopathological examination showed an epithelioid malignant neoplasm with moderate pigmentation and a high mitotic index (11 mitoses/mm²), and immunohistochemistry was positive for SOX10, confirming the diagnosis of primary SNMM. This case highlights the diagnostic challenge of SNMM and emphasizes that persistent unilateral epistaxis refractory to conventional treatment should prompt early endoscopic evaluation to exclude structural lesions, including uncommon sinonasal malignancies.

## Introduction

Primary sinonasal mucosal melanoma (SNMM) is a rare and highly aggressive malignancy [1], with an incidence from 0.02 to 0.149 cases per 100,000 persons per year and an estimated five-year overall survival of only 34.8% (95% CI: 30.6-39.5) [2]. Unlike cutaneous melanoma, its pathogenesis remains poorly understood and is not associated with ultraviolet radiation exposure [3]. The disease is frequently diagnosed at advanced stages because of its nonspecific clinical presentation, resulting in high rates of local recurrence, distant metastasis, and poor survival outcomes [3].

Although a recent systematic review synthesized the available Latin American evidence on primary oral and SNMM, only three of the 33 included studies focused exclusively on sinonasal disease, highlighting the persistent scarcity of site-specific clinicopathological reports [1].

We present a Peruvian case of primary SNMM that adds to the limited Latin American clinicopathological literature on this entity and highlights the diagnostic challenge posed by persistent unilateral epistaxis refractory to conventional management.

## Case presentation

A 70-year-old Peruvian female with a history of hypertension and stroke sequelae presented to the Otolaryngology Department after six months of progressively worsening headache and recurrent epistaxis refractory to conventional treatments, including direct compression, chemical cauterization, and nasal packing. During the same period, she also developed facial pain, foul-smelling nasal discharge, progressive left-sided nasal obstruction, and an unintentional weight loss of approximately 15 kg.

Physical examination revealed right-sided hemiparesis from her previous stroke. Nasofibrolaryngoscopy identified a friable, irregular, and exophytic mass with dark pigmentation protruding from the left nasal cavity. No cervical lymphadenopathy was palpable. A thorough cutaneous and ophthalmological examination ruled out other suspicious melanocytic lesions.

**Figure 1:**
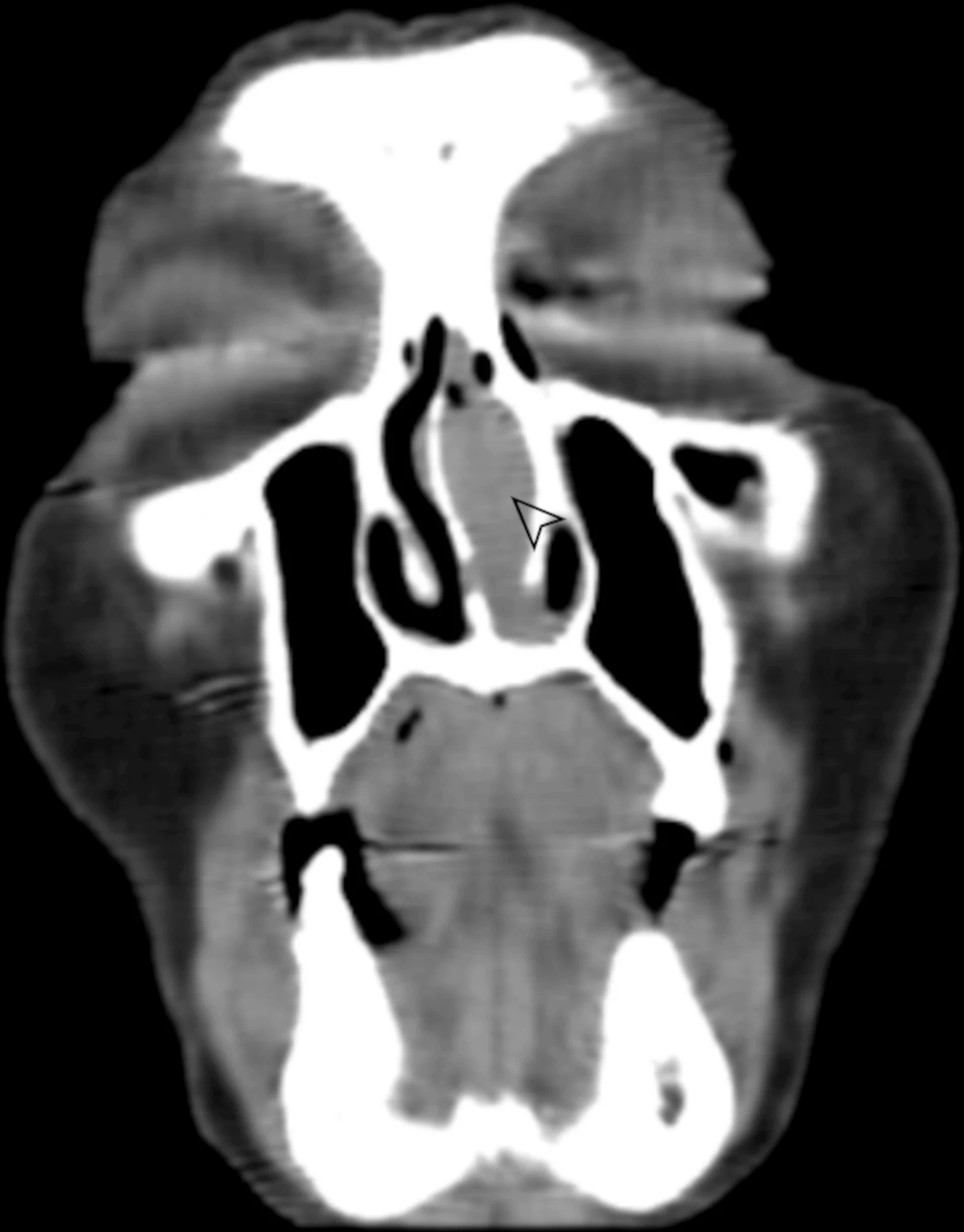
Computed tomography of the paranasal sinuses and nasal cavities (coronal view) Coronal CT image of the paranasal sinuses and nasal cavities showing a hypodense solid mass (39 Hounsfield units (HU) on basal imaging and 44 HU after contrast administration) arising from the left inferior turbinate at the superolateral aspect of the nasal cavity and projecting into the nasal cavity (arrowhead). No evidence of adjacent bone erosion or invasion of the paranasal sinuses or skull base was observed.

**Figure 2:**
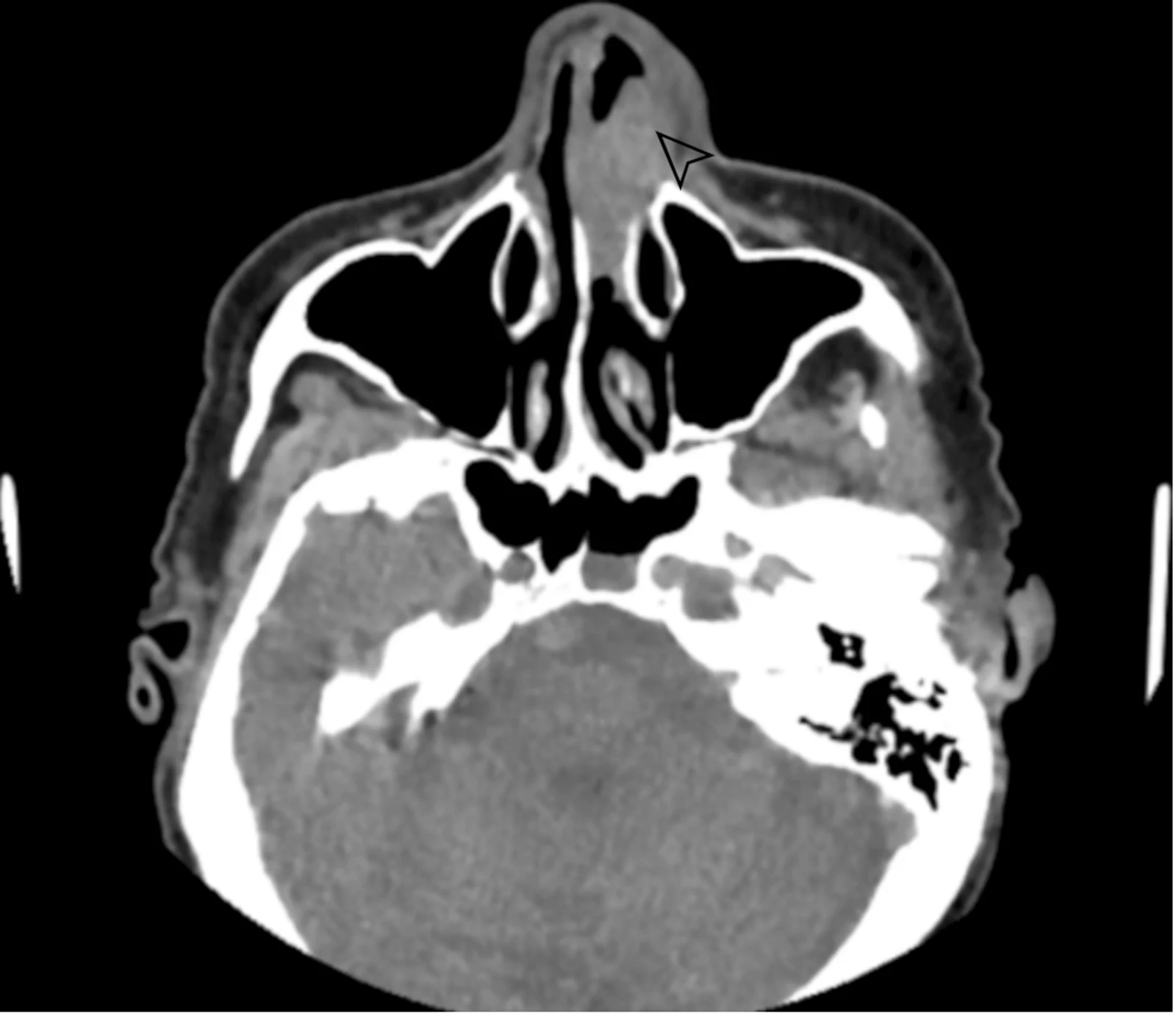
CT of the paranasal sinuses and nasal cavities (axial view) Axial contrast-enhanced CT image showing the same left nasal cavity mass (arrowhead), arising from the inferior turbinate and occupying the nasal cavity. No evidence of adjacent bone destruction or extension to surrounding structures was observed.

A contrast-enhanced computed tomography (CT) of the nose and paranasal sinuses revealed a hypodense mass (39 HU basal, 44 HU post-contrast) arising from the left inferior turbinate and projecting into the nasal cavity. There was no evidence of invasion into the maxillary or ethmoid sinuses or the skull base (Figures 1, 2). Differential diagnoses included nasal polyp, inverted papilloma, and sinonasal malignancy. The patient underwent an initial surgical procedure; however, due to the friability and characteristics of the lesion, complete excision was not feasible, and only an incisional biopsy was obtained.

**Figure 3:**
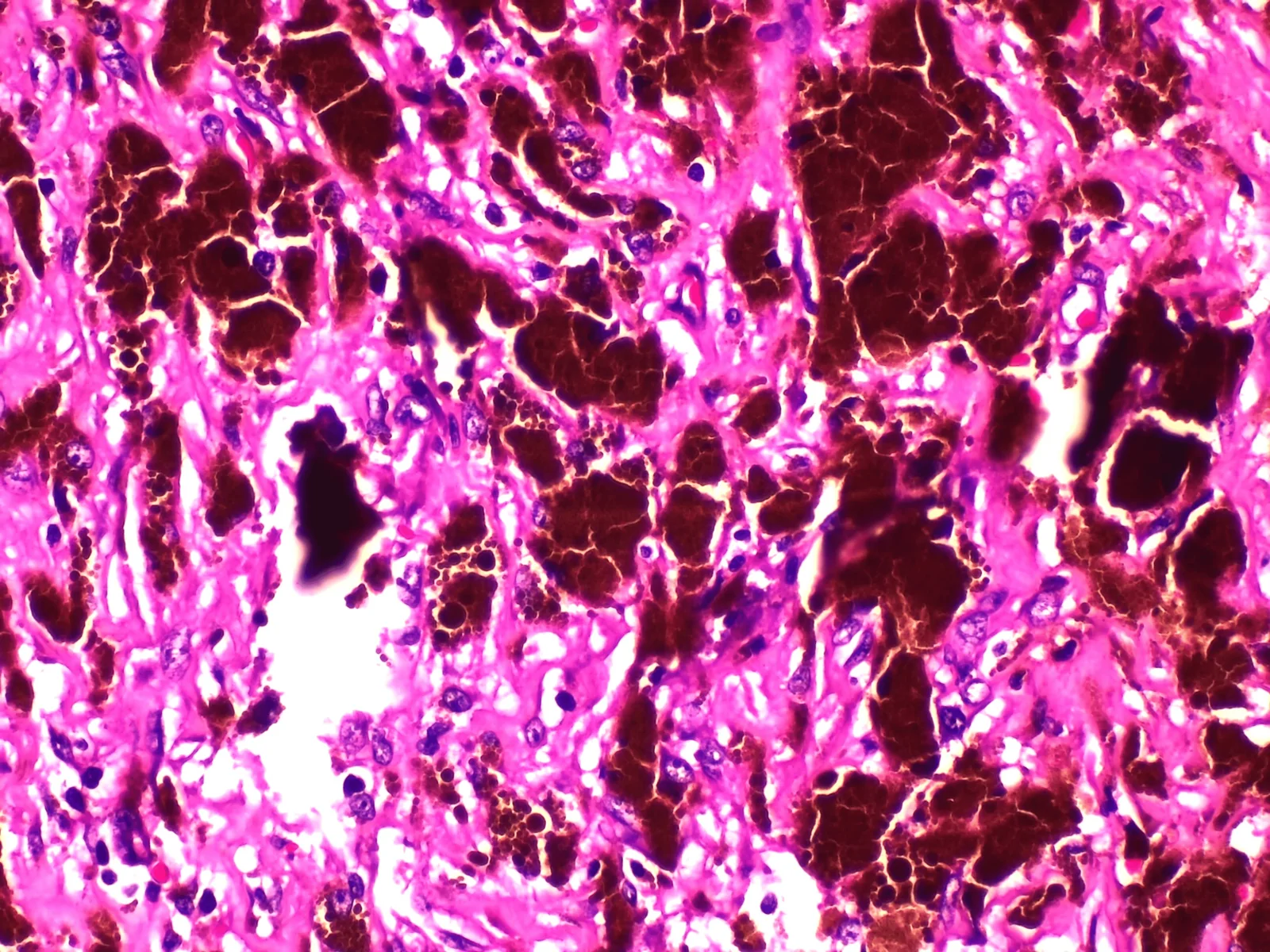
Histopathological examination (hematoxylin and eosin staining, 40× magnification) showing an ulcerated malignant neoplasm composed predominantly of epithelioid cells with melanin pigmentation and increased mitotic activity

**Figure 4:**
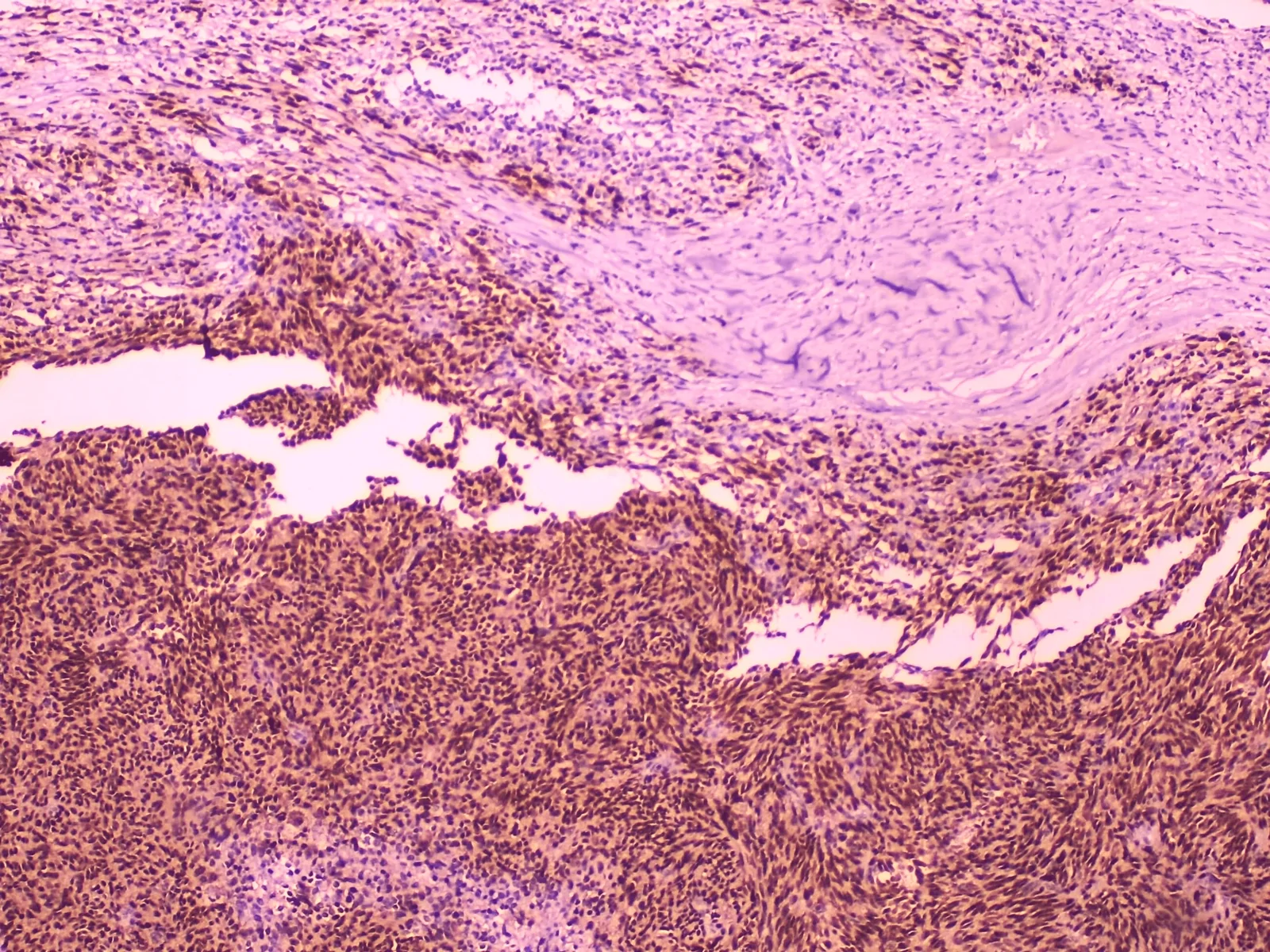
Immunohistochemical staining showing diffuse nuclear positivity for SOX10 in tumor cells, supporting the diagnosis of sinonasal mucosal melanoma

Histopathological examination of the biopsy revealed an ulcerated invasive mucosal melanoma composed predominantly of epithelioid cells with moderate melanin pigmentation and a high mitotic index (11 mitoses/mm²). The specimen was fragmented and extensively infiltrated by the tumor, with involvement of the specimen margins. The largest tissue fragment measured 17 × 8 mm. Vascular and perineural invasion could not be reliably assessed because of specimen fragmentation. Immunohistochemical analysis demonstrated diffuse SOX10 positivity, confirming the diagnosis of invasive SNMM (Figures 3, 4). Additional immunohistochemical markers (S100, HMB-45, and Melan-A) and molecular testing (*KIT*, *BRAF*, and *NRAS*) were not performed because they were not routinely available in our institution, representing a limitation frequently encountered in low-resource settings. 

Following histopathological confirmation, the patient was referred to the Head and Neck Surgical Oncology and Medical Oncology teams for multidisciplinary evaluation and treatment planning. Although initial clinical follow-up was established, subsequent information regarding complete staging, definitive treatment, and long-term outcomes was unavailable at the time of manuscript revision.

## Discussion

Nasal mucosal melanoma represents less than 1% of all melanomas and less than 5% of sinonasal tumors [3]. Unlike cutaneous melanoma, SNMM is not associated with ultraviolet radiation exposure and exhibits a more aggressive clinical course [4]. Epidemiologically, it predominantly affects patients in their seventh and eighth decades of life, with a slight female predilection [5,6]. Geographic, genetic, and environmental factors may affect the presentation and biological behavior of this disease [5].

Clinically, the disease presents with non-specific symptoms such as nasal obstruction, epistaxis, hyposmia, or facial pain [2]. This often leads to confusion with benign lesions, resulting in over 50% of cases being identified at advanced stages [7]. Similar to prior reports, the six-month delay between symptom onset and diagnosis highlights the diagnostic challenge associated with nonspecific sinonasal symptoms, which frequently leads to advanced disease at presentation [2,8]. However, the significant unintentional weight loss of approximately 15 kg observed in our patient may reflect a more pronounced systemic manifestation compared with other reported cases, where symptoms are commonly limited to local sinonasal findings [7].

Diagnostic confirmation relies on clinical evaluation and is established through histopathological and immunohistochemical analyses. SNMMs may present as friable, polypoid nasal masses with variable pigmentation, ranging from dark blue-black to pale yellow in amelanotic variants [7,9].

Given the aggressive behavior and diagnostic complexity of SNMM, current guidelines emphasize the importance of comprehensive staging to guide prognosis and treatment decisions [10,11]. CT and magnetic resonance imaging provide complementary information regarding tumor extension, involvement of critical anatomical structures, and surgical planning, while PET/CT is recommended when regional or distant metastatic disease is suspected, as metastatic status significantly influences prognosis and treatment decisions [10].

Histopathologically, these tumors are characterized by marked heterogeneity, with findings that may include melanin pigmentation, prominent nucleoli, and a high mitotic index [3]. In our patient, the high mitotic activity (11 mitoses/mm²) represented an adverse prognostic feature associated with aggressive tumor behavior and poorer outcomes [4]. Immunohistochemical evaluation is essential for diagnostic confirmation, with markers such as SOX10, S100, HMB-45, and Melan-A (MART-1) supporting melanocytic differentiation, particularly in challenging cases [11,12]. In this case, diffuse SOX10 positivity, together with compatible clinical, imaging, and histopathological findings, supported the diagnosis, although previous reports have described broader immunohistochemical panels including S100, HMB-45, and Melan-A for confirmation [5,7,8].

Molecular profiling is increasingly relevant in SNMM due to its distinct genomic landscape compared with cutaneous melanoma, including more frequent KIT alterations and lower rates of BRAF mutations, potentially allowing targeted therapies in selected patients with advanced disease [9,11,12]. In our patient, molecular profiling was not available. Additionally, complete staging, definitive treatment, and long-term follow-up information were unavailable at the time of manuscript revision due to loss to follow-up after referral for specialized oncologic management.

Surgical resection with negative margins remains the cornerstone of treatment and is often combined with radiotherapy to improve locoregional disease control [8]. More recently, immunotherapy, particularly immune checkpoint inhibitors targeting the PD-1 and CTLA-4 pathways, has emerged as a promising therapeutic option for patients with advanced disease [9]. Nevertheless, SNMM remains one of the most aggressive melanoma subtypes, with a significantly poorer prognosis than cutaneous melanoma. This unfavorable outcome is largely attributable to its anatomical location, which hinders early detection [3], as well as its lower responsiveness to conventional therapies [9].

The estimated five-year overall survival rate is approximately 34.8% (95% CI: 30.6-39.5) [2], reflecting the high rates of local recurrence and distant metastasis associated with this malignancy [8]. In this context, early diagnosis and a multidisciplinary approach are essential to optimize clinical outcomes and improve patient survival.

Recent evidence has improved the characterization of mucosal melanoma in Latin America; however, reports dedicated specifically to SNMM remain uncommon [1]. Individual well-documented cases continue to be valuable because they strengthen the regional evidence base, facilitate future collaborative analyses, and improve recognition of this uncommon malignancy in routine clinical practice.

Beyond its geographic origin, this case emphasizes an important clinical lesson. Persistent unilateral nasal bleeding that fails to respond to conventional measures should prompt early evaluation to exclude structural lesions, including uncommon sinonasal malignancies such as mucosal melanoma. Earlier recognition may reduce diagnostic delay and facilitate timely multidisciplinary management.

## Conclusions

SNMM remains a rare and highly aggressive malignancy whose diagnosis is frequently delayed because of its nonspecific clinical presentation. This case highlights the importance of recognizing alarm features such as recurrent unilateral epistaxis, facial pain, and unexplained weight loss, particularly in elderly patients. The presence of epithelioid morphology and a high mitotic index (11 mitoses/mm²) suggested an aggressive biological phenotype despite the absence of radiological evidence of local invasion.

This case adds to the limited Latin American literature specifically addressing SNMM and reinforces the importance of recognizing unilateral nasal bleeding that persists despite conventional therapy as a warning sign requiring prompt endoscopic evaluation. Continued reporting of well-characterized regional cases remains essential to strengthen collaborative evidence and improve the understanding of this uncommon malignancy.
